# Supplementary material for: Does a specialized assessment improve vocational outcomes for people on sick leave with a suspected common mental disorder? Results from the Mental Health Assessment Study (MeHAS)
Source: PLOS Ment Health. 2024 Jun 4;1(1):e0000020. doi: 10.1371/journal.pmen.0000020 (PMC12798238; doi:10.1371/journal.pmen.0000020)
Supplement: S1 File — (DOCX) [file pmen.0000020.s001.docx]

Protocol deviations

This supplement is sought to be an exhaustive listing of deviations from protocol in The Mental Health Assessment Study.

| A priori protocol plan | Deviation | Researchers’ intentionality | Reason for deviation |
| --- | --- | --- | --- |
| *This column describes the original, pre-registered plan* | *This column describes what actually happened* | *This column describes whether the deviation was intentional from the researchers, or e.g., due to poor implementation* | *This column: If the deviation was intentional, the rationale is provided; if not intentional, the speculated reason for deviation is provided* |
| Allocation to interventions were planned to be totally random, and hence the preregistered title “a quasi-randomized trial” | In practice, the staff who referred participants into inclusion and allocation might have been able to predict likelihood of allocation, depending on certain variables, like weekday, time of month and time of day. For this reason, we describe the study as an experimental study. | This change was due to unintended poor implementation of the protocol | In practice, it was not feasible for the collaboration municipality to perform the stricter randomizing procedure that was planned. |
| Employment status was not listed in eligibility criteria. | Only those recruited who were on sick-leave due from work, and not from vacancy, were included iwn the analyses. | We intentionally, post-hoc excluded participants on sick leave from vacancy, including only employed participants. | Participants on sick leave from vacancy were recruited through a specific municipal office, managing vacancy cases. We learned that in this office, the case managers who recruited participants, usually could predict allocation from time of day. Hence a substantial risk of bias was introduced. |
| Baseline date should be date of intervention allocation | For all cases, we imputed baseline date, as being the date 21 days after first day of sick leave. Day of first sick-leave we calculated by finding the first long-term sickness period in the recruitment period. | We intentionally, post-hoc, decided to use this method instead, since the actual allocation dates were not recorded in the control group, and hence had to be imputed. | Due to poor implementation, allocation date nor first day of sick leave was not registered (in the control group), and hence was missing.  In the intervention group we observed the first day of sick leave, but not allocation date. We used the same imputation method in both groups, despite we observed day of first sick-leave in the intervention group. Yet, we preferred introducing an equal amount of possible deviation in both groups, in order to reduced bias of the estimates of group differences. |
| An inclusion criterion was “any mental health disorder established of suspected as the main cause of sick-leave” | In practice a non-planned exclusion criterion was implicitly implemented: If the absentee by the jobcentre staff was deemed to have low probability of being eligible to one of two concurrent RCTs, the were often not allocated despite protocol. | This change was due to poor implementation of protocol, not intended. | We speculate that in practice, case managers did not find it beneficial (and hence not ethical) to be delayed by the mental health assessment (the intervention in this study), why they did not comply with protocol. |

### Post-hoc sensitivity analyses

Post-hoc we decided to conduct two sensitivity analyses: 1) We decided to explore the robustness of the results by performing the analyses on participants who three weeks after baseline was still on sick leave only. We suspected that in the intervention group, since the assessment usually implied waiting time of up to three weeks after referral, they were not likely to return to work until hereafter, and we wanted to examine if any differences in main analyses would remain after this time period; 2) We also decided to perform the analyses excluding participants who did not complete the assessment, being a complete-case analysis.
