# Supplementary material for: Does a specialized assessment improve vocational outcomes for people on sick leave with a suspected common mental disorder? Results from the Mental Health Assessment Study (MeHAS)
Source: PLOS Ment Health. 2024 Jun 4;1(1):e0000020. doi: 10.1371/journal.pmen.0000020 (PMC12798238; doi:10.1371/journal.pmen.0000020)
Supplement: S2 File — (DOCX) [file pmen.0000020.s002.docx]

Results

# Post-hoc analyses

## Post-hoc sensitivity analyses

Regarding the analyses of only participants still on sick leave three weeks after baseline, the differences in proportion in work at follow-up were slightly larger, but the difference in RTW hazards rates were no longer observed. See Table B

Regarding the complete case-sensitivity analyses, the results were similar, yet with slightly bigger differences and lower p-values. One outcome, time to RTW (full-time definition) changed from HR=0.88 (p=0.055) to the statistically significant HR=0.84 (p=0.013). See Table C.

### Analyses of diagnostic distribution

Among the entire assessed population, stress-related disorders (including adjustment disorders) were most frequent (n=495, 38.9%), followed by depression (n=383, 30.1%). Most of the assessed participants (86.5%) were previously diagnosed at their general practitioner, yet some with a combination of diagnoses (e.g. “stress, anxiety and depression”), but some of these diagnoses did not align with the ones at the assessment in this study. E.g., of 608 participants referred with stress, 139 were found to have depression and 71 anxiety, in total 35%. Of the 492 found to have stress in combination with depression or anxiety, only 138 (28%) were found to have only stress-related disorders. See Fig A.

| Variable | Value | MeHAS intervention | Control group | p |
| --- | --- | --- | --- | --- |
|  | n (%) | 895 (100) | 1048 (100) | |
| n (%, weighted) | | 429 (100) | 1048 (100) | |
| Sex | Female | 312 (72.7) | 704 (67.2) | 0.04 |
|  | Male | 117 (27.3) | 344 (32.8) |  |
| Age | Mean years (SD) | 40.51 (10.4) | 40.05 (10.5) | 0.426 |
| Education | 1. Primary school | 97 (22.6) | 237 (22.6) | 0.211 |
|  | 2. Secondary and vocational | 125 (29.1) | 277 (26.4) |  |
|  | 3. proff/academic | 77 (17.9) | 239 (22.8) |  |
|  | N/A | 130 (30.3) | 295 (28.1) |  |
| Civic status | 1. Not married/cohabitating | 238 (55.5) | 530 (50.6) | 0.098 |
|  | 2. Married/cohabitating | 191 (44.5) | 518 (49.4) |  |
| Employment status | 1. Self employed | 6 (1.4) | 25 (2.4) | 0.395 |
|  | 2. Salaried manager | 76 (17.7) | 212 (20.2) |  |
|  | 3. Salaried worker | 133 (31) | 293 (28) |  |
|  | 4. Income transfer | 53 (12.4) | 118 (11.3) |  |
|  | 5. In education | 161 (37.5) | 400 (38.2) |  |
| Weeks with sickness benefit before baseline | | 9.34 (12.89) | 9.03 (13.24) | 0.344 |
| Weeks with any benefit and no salary before baseline | | 16.25 (27.15) | 19.84 (30.45) | 0.079 |
| Employment before sick leave, n (%) | | 351 (81.8) | 756 (72.1) | >0.0005 |
| BAI (anxiety) | | 19.96 (10.54) | n/a |  |
| BDI (depression) | | 24.95 (10.94) | n/a |  |
| WSAS (functioning) | | 23.87 (8.67) | n/a |  |
| PSS (distress) | | 24.51 (6.31) | n/a |  |
| IPQ (self-efficacy) | | 14.78 (3.7) | n/a |  |
| QOLS (life quality) | | 60.44 (14.32) | n/a |  |
| KES (exhaustion) | | 83.04 (15.99) | n/a |  |
| GSS (self-efficacy) | | 23.1 (6.84) | n/a |  |
| 4DSQ (somatization) | | 15.07 (9.14) | n/a |  |
| 4DSQ (distress) | | 20.02 (7.96) | n/a |  |
| 4DS (anxiety) | | 7.25 (6.23) | n/a |  |
| 4DSQ (depression) | | 3.73 (3.55) | n/a |  |
| RTW-SE (RTW self-efficacy) | | 13.73 (7.96) | n/a |  |
| EQ5 (life quality) | | 0.67 (0.17) | n/a |  |
| ASRS (ADHD symptoms) | | 8.06 (4.68) | n/a |  |
| SAPAS (personality disorder screening) | | 2.81 (1.27) | n/a |  |

Table A: Baseline values of the included population (before exclusion of persons on sick leave from unemployment). ); BDI: Bech Depression Inventory; BAI: Bech Anxiety Inventory; PSS: Perceived Stress Scale; WSAS: Work and Social Adjustment Scale; 4DSQ: Four Dimensional Questionnaire; KES: Karolinska exhaustion disorder scale; IPQ: Illness Perception Questionnaire; EQ5DL: Health related quality of life; QoLs: Quality of Life Scale; RTW-SE: Return to work-self efficacy; SPS: Stepford Presenteeism scale; GSE: Generalized Self-Efficacy Scale; SAPAS: Assessment of Personality – Abbreviated Scale; ASRS: Attention deficit hyperactivity disorder symptom checklist for adults.

|  | Group values | | Group comparisons | |
| --- | --- | --- | --- | --- |
|  | Control group | Intervention group | Estimate | p-val. |
| n (%) | 726 (100) | 686 (100) |  |  |
| Full-time work status at  12 months. n (%) | 439 (60.5) | 179 (54.1) | 0.77 [OR]  (CI: 0.59 to 1.00) | 0.052 |
| Part- or full-time work status at  12 months, n (%) | 458 (63.1) | 186 (56.2) | 0.75 [OR]  (CI: 0.58 to 0.98) | 0.034 |
| Weeks in full time work  during 12 months (SD) [median] | 23.5 (17.7) [27] | 20.1 (16.0) [22] | 0.85 [RR]  (CI: 0.78 to 0.93) | <0.001 |
| Weeks in part- or full-time work  during 12 months (SD) [median] | 30.4 (20.9) [38] | 25.0 (19.6) [27] | 0.82 [RR]  (CI: 0.76 to 0.90) | <0.001 |
| Sick leave duration before full-time work, days (SD) [median] | 156.3 (122.4) [119] | 172.4 (123.8) [154] | 0.84 [HR]  (CI: 0.74 to 0.96) | 0.009 |
| Sick leave duration before part- or full-time work, days (SD) [median] | 173.7 (153.6) [105] | 167.8 (144.7) [126] | 1.09 [HR]  (CI: 0.94 to 1.25) | 0.245 |

Table B: Vocational outcomes of sensitivity analyses, only participants still on sick-leave three weeks after baseline included, at 12-month follow-up; SD: Standard Deviation; HR: Hazard Ratio; n/a: not available; CI: confidence interval; RR: Relative Risk; OR: Odds Ratio

|  | Group values | | Group comparisons | |
| --- | --- | --- | --- | --- |
|  | Control group | Intervention group | Estimate | p-val. |
| n (%) | 756 (100) | 451 (100) |  |  |
| Full-time work status at 12 months  n (%) | 444 (58.7) | 142 (55.3) | 0.87 [OR]  (CI: 0.66 to 1.16) | 0.348 |
| Part- or full-time work status,  12 months, n (%) | 458 (60.6) | 147 (57.2) | 0.87 [OR]  (CI: 0.65 to 1.16) | 0.340 |
| Weeks in full time work  during 12 months (SD) [median] | 21.3 (16.5) [23] | 17.7 (14.3) [19] | 0.83 [RR]  (CI: 0.75 to 0.92) | <0.001 |
| Weeks in part- or full-time work  during 12 months (SD) [median] | 28.0 (20.1) [33.5] | 22.9 (18.5) [24] | 0.82 [RR]  (CI: 0.74 to 0.90) | <0.001 |
| Sick leave duration before full-time work, days (SD) [median] | 170.8 (118.9) [140] | 191.1 (117.0) [175] | 0.84[HR]  (CI: 0.73 to 0.96) | 0.013 |
| Sick leave duration before part- or full-time work, days (SD) [median] | 120.8 (127.9) [63] | 156.2 (133.6) [105] | 0.76 [HR]  (CI: 0.66 to 0.87) | <0.001 |

Table C: Vocational outcomes (complete case sensitivity analyses), at 12-month follow-up; SD: Standard Deviation; HR: Hazard Ratio; n/a: not available; CI: confidence interval; RR: Relative Risk; OR: Odds Ratio

| Service type | Specific intervention measure | | MeHAS-intervention | Control group | p-value |
| --- | --- | --- | --- | --- | --- |
| Register data: Mental health care | **Sessions, GP** | Mean (SD); Median | 8.42 (5.6); 7 | 7.65 (5.3); 6 | 0.023 |
|  | **Sessions, psychologist** | Mean (SD); Median | 0.81 (2.5); 0 | 0.61 (2.2); 0 | 0.153 |
|  | **Sessions, psychiatrist** | Mean (SD); Median | 0.40 (1.7); 0 | 0.36 (1.5); 0 | 0.569 |
|  | **≥ 1 out-patient psych. contact** | n (proportion, [%]) | 62 (18.0) | 99 (13.1) | 0.039 |
|  | **≥ 1 psych. admission** | n (proportion, [%]) | 33 (9.5) | 85 (11.2) | 0.459 |
|  | **≥ 1 psych. ER contact** | n (proportion, [%]) | 0 (0.0) | 0 (0.0) | N/A |
| Register data: Vocational rehabilitation | **EC meetings** | Mean (SD); Median | 2.67 (1.9); 2 | 1.92 (1.9); 1 | 0.000 |
|  | **EC virtual contacts** | Mean (SD); Median | 1.73 (1.7); 1 | 2.08 (1.7); 2 | 0.001 |
|  | **VR course** | n (proportion [%]) | 87 (25.1) | 125 (16.5) | 0.001 |
|  | **VR course, hours (cumulated)** | Mean (SD); Median | 13.1 (33.5); 0 | 8.13 (24.0); 0 | 0.001 |
|  | **VR course duration, days (start-to-end)** | Mean (SD); Median | 13.5 (29.2); 0 | 8.53 (23.9); 0 | 0.001 |

Table D: Interventions delivered from Baseline to 12-month follow-up; GP: General Practitioner; VR: Vocational Rehabilitation; EC: Employment Consultant; SD: Standard Deviation; ER: Emergency Room; n/a: not available

Fig A: Flow between referral and assessment diagnoses (entire assessed population); at assessment “stress” includes adjustment disorders (F43).


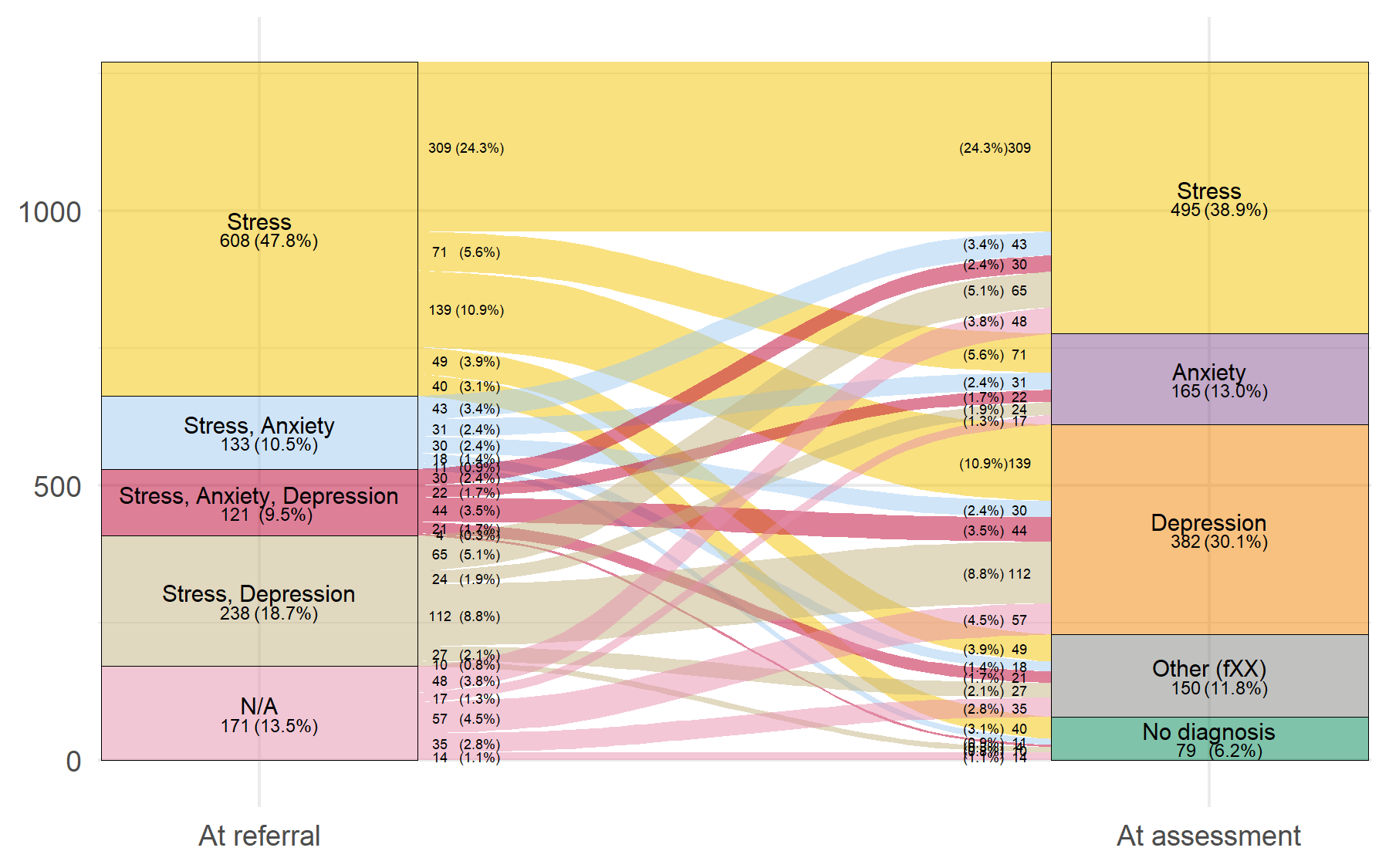


At referral

At assessment
