# Supplementary material for: Does a specialized assessment improve vocational outcomes for people on sick leave with a suspected common mental disorder? Results from the Mental Health Assessment Study (MeHAS)
Source: PLOS Ment Health. 2024 Jun 4;1(1):e0000020. doi: 10.1371/journal.pmen.0000020 (PMC12798238; doi:10.1371/journal.pmen.0000020)
Supplement: Attachment — Submitted filename: Response to Reviewers.docx [file pmen.0000020.s003.docx]

Rebuttal letter

- regarding the revised paper “DOES A SPECIALIZED ASSESSMENT IMPROVE VOCATIONAL OUTCOMES FOR PEOPLE ON SICK LEAVE WITH A SUSPECTED COMMON MENTAL DISORDER? RESULTS FROM THE MENTAL HEALTH ASSESSMENT STUDY (MEHAS)”

Copenhagen, January 2024

Dear editor

Thank you very much for your kind response to our paper.

# Regarding editorial remarks

Regarding data sharing: We hold the value of data sharing extremely high, and have already collaborated with other researched who wanted e.g. to validate a Danish version of their (Dutch) questionnaire. Hence, we have tried to clarify and emphasize that we would happily do all we can to ensure data sharing. If it was not clear in the submitted manusscript, we apologize! Not only do we indicate willingness to share data due to PLOS’ policies, but also because we believe it is a basic scientific virtue to do so!

Regarding references: We have reviewed the reference list, and have corrected a reference to a report with a Danish title, and it now appears with English translation.

The Supplements now have a legend.

# Reviewers’ comments

Regarding informed consent: We have somewhat heavily revised the section “Allocation” to clarify exactly how the collection of informed consent was carried out in the study, as seen in the revised paper where changes are tracked.

Regarding procedures: In the sub-chapter “Interventions” under “Intervention group” we have elaborated how the instruments used were applied.

Regarding implications: In the first submitted version of the paper, we perhaps were too humble with regards to what the paper’s implications should be. The reviwers’ comments have inspirered us to be clearer, so we have elaborated both what the implications for policies and for future research are.

On behalf of all authors

Andreas Hoff, PhD
